# Generation of intense dissipation in high Reynolds number turbulence

**DOI:** 10.1098/rsta.2021.0088

**Published:** 2022-03-07

**Authors:** Dhawal Buaria, Alain Pumir, Eberhard Bodenschatz

**Affiliations:** ^1^ Tandon School of Engineering, New York University, New York, NY 11201, USA; ^2^ Max Planck Institute for Dynamics and Self-Organization, Göttingen 37077, Germany; ^3^ Laboratoire de Physique, ENS de Lyon and CNRS, Lyon 69007, France; ^4^ Institute for Nonlinear Dynamics, University of Göttingen, Göttingen 37077, Germany

**Keywords:** turbulence, intermittency, rare events, Navier–Stokes equations, nonlinear amplification

## Abstract

Intense fluctuations of energy dissipation rate in turbulent flows result from the self-amplification of strain rate via a quadratic nonlinearity, with contributions from vorticity (via the vortex stretching mechanism) and pressure-Hessian—which are analysed here using direct numerical simulations of isotropic turbulence on up to $12\;288^{3}$ grid points, and Taylor-scale Reynolds numbers in the range 140–1300. We extract the statistics involved in amplification of strain and condition them on the magnitude of strain. We find that strain is self-amplified by the quadratic nonlinearity, and depleted via vortex stretching, whereas pressure-Hessian acts to redistribute strain fluctuations towards the mean-field and hence depletes intense strain. Analysing the intense fluctuations of strain in terms of its eigenvalues reveals that the net amplification is solely produced by the third eigenvalue, resulting in strong compressive action. By contrast, the self-amplification acts to deplete the other two eigenvalues, whereas vortex stretching acts to amplify them, with both effects cancelling each other almost perfectly. The effect of the pressure-Hessian for each eigenvalue is qualitatively similar to that of vortex stretching, but significantly weaker in magnitude. Our results conform with the familiar notion that intense strain is organized in sheet-like structures, which are in the vicinity of, but never overlap with tube-like regions of intense vorticity due to fundamental differences in their amplifying mechanisms.

This article is part of the theme issue ‘Scaling the turbulence edifice (part 1)’.

## 1. Introduction

The dissipation rate of kinetic energy, ϵ, defined as

1.1 $$\epsilon =2\nu S_{ij}S_{ij},\;\text{where}\;S_{ij}=\frac{1}{2}\left(\frac{\partial u_{i}}{\partial x_{j}}+\frac{\partial u_{j}}{\partial x_{i}}\right),$$

plays an indispensable role in our understanding of turbulent fluid flows. Here, ν is the kinematic viscosity and $S_{ij}$ is the strain rate tensor (the symmetric part of the velocity gradient tensor $\partial u_{i}/\partial x_{j}$). The mean of dissipation rate quantifies the net cascade of energy from large to small scales, manifestly becoming independent of ν, as ν→0 [1–3]. This property, also known as dissipative anomaly, is the central tenet of essentially all turbulence theories and models [1]. However, the fluctuations of dissipation rate (and hence that of strain rate) can be orders of magnitude larger than its mean [4,5], a phenomena known as intermittency, which renders any mean-field description of turbulence inadequate [1,6]. Understanding the formation of such intense fluctuations and characterizing their statistical properties has long remained one of the outstanding challenges in turbulence [1,7].

Understanding the intense fluctuations of dissipation is also directly important from a practical standpoint. For instance, strong strain rates can greatly enhance dispersion of particles and influence mixing of scalars or can adversely affect flame propagation in reacting flows [8–11]. Intense strain also leads to generation of intense vorticity, via the well-known vortex stretching mechanism [12], which in turn influences clustering of inertial particles [13]. In fact, strain and vorticity are not independent and their coupling implicitly encodes all the multiscale interactions in the flow [14,15]. While much attention has been recently given to understand this interaction in the light of vorticity amplification [15–17] and energy cascade across scales [18,19], in the current work, we present a complementary investigation focusing on amplification of strain (and hence dissipation rate).

The key mechanisms controlling amplification of strain can be readily identified by writing its transport equation (as derived from the incompressible Navier–Stokes equations):

1.2 $$\frac{DS_{ij}}{Dt}=-S_{ik}S_{kj}-\frac{1}{4}(\omega_{i}\omega_{j}-\omega_{k}\omega_{k}\delta_{ij})-\Pi_{ij}+\nu \nabla^{2}S_{ij},$$

where ω=∇×u is the vorticity vector and $\Pi_{ij}=(1/\rho )(\partial^{2}P/\partial x_{i}\partial x_{j})$ is the pressure Hessian tensor. The first term on the r.h.s. of equation (1.2) captures the self-amplification of strain, which by itself could lead to a finite time singularity. The second term captures the influence of vorticity and essentially the feedback of vortex stretching on strain itself. The third term involving pressure-Hessian represents the influence of non-local effects via the pressure field, and hence couples the entire state of the flow. This nonlocal dependence is readily seen by taking the trace of equation (1.2), leading to the Poisson equation

1.3 $$\Pi_{ii}=\frac{\nabla^{2}P}{\rho }=\frac{\left(\omega_{i}\omega_{i}-2S_{ij}S_{ij}\right)}{2}.$$

The final (linear) term in equation (1.2) represents the viscous diffusion of strain.

Here, our main goal is to investigate various amplification mechanisms leading to the formation of intense strain and hence dissipation. To this end, we analyse the statistics of the (inviscid) nonlinear terms in equation (1.2), in particular by conditioning them on magnitude of strain. One of the objectives is to identify and understand which (non-viscous) mechanism(s) help to prevent an unbounded growth of strain [15]. We use data from high-resolution direct numerical simulations (DNS) of isotropic turbulence in periodic domains, which is the most efficient numerical tool to study the small-scale properties of turbulence. Another important purpose of the current study is also to understand the effect of increasing Reynolds number. With that in mind, we use a massive DNS database with Taylor-scale Reynolds number $R_{\lambda }$ ranging from 140 to 1300 on grid sizes going up to $12\;288^{3}$. These simulations have particularly high small-scale resolution to accurately capture the extreme fluctuations [15–17].

To get insight into the formation of intense strain, we compute various statistics related to strain amplification, conditioned on magnitude of strain. We find that the self-amplification solely drives the growth of intense fluctuations, whereas vortex stretching and pressure Hessian terms act to attenuate this growth. By decomposing various contributions in the eigenframe of strain tensor, we further show that this amplification and attenuation predominantly occurs for the most negative eigenvalue, signifying intense strain events correspond to strong compressive motion. By contrast, the other two eigenvalues are amplified by the vortex stretching mechanism and depleted by the self-amplification term, with both these mechanisms canceling each other out almost perfectly. The effect of the pressure Hessian, qualitatively similar to that of vortex stretching, is to weakly amplify these two eigenvalues. The structure of the nonlinearities uncovered in this work is consistent with the general notion that regions of intense strain are organized in sheet-like structures [20,21], which are unlikely to be colocated with regions of intense vorticity organized in tube-like structures [22,23]—underscoring the importance of non-local interactions between strain and vorticity in amplifying gradients [15,17].

The rest of the manuscript is organized as follows. In §2, we briefly provide the details of the DNS database used in this work. The various nonlinearities controlling the amplification of strain are presented in §3, in particular by analysing their statistics on magnitude of strain. In §3(c), the various contributions are further analysed in the eigenbasis of strain tensor. Finally, we summarize our results in §4.

## 2. Numerical approach and database

The data used here are the same as in recent works [5,15–17,24] and obtained from DNS of incompressible Navier–Stokes equations, for the canonical setup of isotropic turbulence in a periodic domain. The simulations are carried out using highly accurate Fourier pseudo-spectral methods with second-order Runge–Kutta integration in time, and the large scales are forced numerically to achieve statistical stationarity. A key characteristic of our data is that we have achieved a wide range of Taylor-scale Reynolds number $R_{\lambda }$, going from 140 to 1300, while maintaining excellent small-scale resolution on grid sizes going up to $12\;288^{3}$. The resolution is as high as $k_{\max}\eta \approx 6$, where $k_{\max}=\sqrt{2}N/3$ is the maximum resolved wavenumber on a $N^{3}$ grid, and η is the Kolmogorov length scale. The spatial resolution of the $R_{\lambda }=1300$ run is less than for the runs at lower $R_{\lambda }$, and statistics have been accumulated over a comparatively shorter time, in units of $T_{E}$. Nonetheless, convergence with respect to resolution and statistical sampling has been adequately established in previous works [15,16]. We summarize the DNS database and the simulation parameters in table 1.

**Table 1.** Simulation parameters for the DNS runs used in the current work: the Taylor-scale Reynolds number ($R_{\lambda }$), the number of grid points ($N^{3}$), spatial resolution ($k_{\max}\eta$), ratio of large-eddy turnover time ($T_{E}$) to Kolmogorov time scale ($\tau_{K}$), length of simulation ($T_{\mathrm{sim}}$) in statistically stationary state and the number of instantaneous snapshots ($N_{s}$) used for each run to obtain the statistics.

$R_{\lambda }$	$N^{3}$	$k_{\max}\eta$	$T_{E}/\tau_{K}$	$T_{\mathrm{sim}}$	$N_{s}$
140	$1024^{3}$	5.82	16.0	$6.5T_{E}$	24
240	$2048^{3}$	5.70	30.3	$6.0T_{E}$	24
390	$4096^{3}$	5.81	48.4	$2.8T_{E}$	35
650	$8192^{3}$	5.65	74.4	$2.0T_{E}$	40
1300	$12\;288^{3}$	2.95	147.4	$20\tau_{K}$	18

## 3. Statistics conditioned on strain/dissipation

In order to quantify the intensity of strain, we consider the quantity Σ defined as

3.1 $$\Sigma =2S_{ij}S_{ij},$$

which is simply the dissipation rate without the viscosity, i.e. Σ=ϵ/ν. The benefit of directly using Σ is that its mean defines the Kolmogorov time scale $\tau_{K}$, i.e. $\langle \Sigma \rangle =1/\tau_{K}^{2}$ and from homogeneity is also equal to the mean of enstrophy, i.e. ⟨Σ⟩=⟨Ω⟩, where $\Omega =\omega_{i}\omega_{i}$. From equation (1.2), the following transport equation for Σ can be derived

3.2 $$\frac{1}{4}\frac{D\Sigma }{Dt}=-S_{ij}S_{jk}S_{ki}-\frac{1}{4}\omega_{i}\omega_{j}S_{ij}-S_{ij}\Pi_{ij}+\nu S_{ij}\nabla^{2}S_{ij},$$

where the term $\omega_{i}\omega_{j}S_{ij}$, which leads to production of enstrophy when considering vorticity transport equation [16], clearly demonstrates the feedback of vortex stretching on amplification of strain.

In statistically stationary isotropic turbulence, as considered in this work, the mean of the l.h.s. of equation (3.2) is zero. For the terms on r.h.s. it is known that [7,25]

3.3 $$\langle S_{ij}\Pi_{ij}\rangle =0$$

and

3.4 $$-\langle S_{ij}S_{jk}S_{kj}\rangle =\frac{3}{4}\langle \omega_{i}\omega_{j}S_{ij}\rangle .$$

Using these relations, the averaging of equation (3.2) leads to

3.5 $$\langle S_{ij}S_{jk}S_{ki}\rangle =\frac{3}{2}\nu \langle S_{ij}\nabla^{2}S_{ij}\rangle ,$$

which gives a simple balance between inviscid production and viscous dissipation of strain. Since $\left\langle \omega_{i}\omega_{j}S_{ij}\right\rangle$ is known to be positive on average [25], it follows that $-\langle S_{ij}S_{jk}S_{kj}\rangle >0$, therefore implying generation of strain via a self-amplification mechanism. While the results in equations (3.4) and (3.5) hold on average for the entire flow field, they do not imply any particular relation when considering the same statistics conditioned on Σ (which is required to isolate the extreme events from the mean-field). In the following, we investigate the role of various terms in equation (3.2) conditioned on Σ.

### (a) Strain self-amplification and vortex stretching

**Figure 1.**
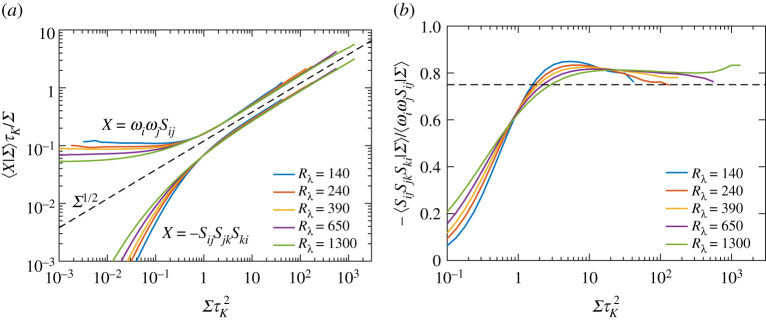
(*a*) Conditional expectations (given Σ) of the strain self-amplification and vortex stretching terms, for various $R_{\lambda }$. All quantities are normalized by Kolmogorov time scale $\tau_{K}={\left\langle \Sigma \right\rangle }^{-1/2}$. The black dashed line indicates the power-law $\Sigma^{1/2}$. (*b*) The ratio of conditional strain self-amplification and vortex stretching terms. The horizontal dashed line indicates the value 3/4, corresponding to the ratio of unconditional averages as given by equation (3.4). (Online version in colour.)

In this subsection, we analyse the contributions from the self-amplification and vortex stretching terms when conditioned on magnitude of strain, i.e. ⟨X|Σ⟩, with respectively $X=-S_{ij}S_{jk}S_{ki}$ and $X=\omega_{i}\omega_{j}S_{ij}$. Figure 1*a* shows both terms, divided by Σ for convenience and for various $R_{\lambda }$. All quantities are appropriately non-dimensionalized by $\tau_{K}$, which allows us to demarcate the strength of events with respect to the mean-field. The main observation is that both the plotted quantities in figure 1*a* scale as $\Sigma^{1/2}$ (marked by black dashed line) for events stronger than the mean ($\Sigma \tau_{K}^{2}≳1$), which implies the conditional expectations themselves scale as $\Sigma^{3/2}$—consistent with simple dimensional argument. Moreover, the dependence on $R_{\lambda }$ is very weak (especially as $R_{\lambda }$ increases), suggesting an asymptotic state has likely been reached.

It should be noted that the magnitude of the vortex stretching term (which depletes strain) is larger, but its net contribution is still lower than the self-amplification term due to the factor of 1/4 in equation (3.2). To further investigate their relative contributions, figure 1*b* shows the ratio of their conditional expectations for various $R_{\lambda }$. For extreme events of strain, we observe that the ratio seemingly asymptotes to a constant value of about 0.8, which is different and slightly larger than 3/4, the value of their unconditional averages. This implies that the overall (negative) contribution of the vortex stretching term is about 1/(4×0.8)≈1/3.2 times that of the strain self-amplification term, which is slightly smaller than the factor 1/3 valid for the overall field (as seen from equations (3.2) and (3.4)).

To better understand the results in figure 1, we analyse them further in the eigenframe of strain, as given by its three eigenvalues $\lambda_{i}$ for i=1,2,3, (such that $\lambda_{1}\geq \lambda_{2}\geq \lambda_{3}$) and the corresponding eigenvectors $e_{i}$. Incompressibility imposes $\lambda_{1}+\lambda_{2}+\lambda_{3}=0$, which renders $\lambda_{1}$ to be always positive (stretching) and $\lambda_{3}$ to be always negative (compressive). It is well known that the second (intermediate) eigenvalue $\lambda_{2}$ is positive on average, leading to net production of enstrophy [16,25,26]. Using the eigenframe, we can readily show that

3.6 $$\Sigma =2(\lambda_{1}^{2}+\lambda_{2}^{2}+\lambda_{3}^{2})\;\text{and}\;S_{ij}S_{jk}S_{ki}=\lambda_{1}^{3}+\lambda_{2}^{3}+\lambda_{3}^{3}=3\lambda_{1}\lambda_{2}\lambda_{3}.$$

**Figure 2.**
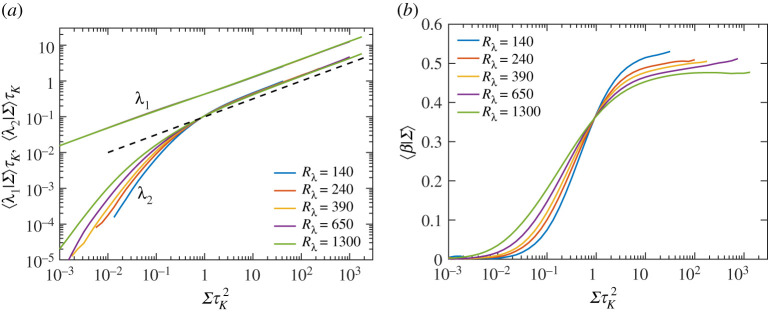
Conditional expectations (given Σ) of (*a*) first two eigenvalues of strain tensor, (*b*) the ratio β as defined by equation (3.7), for various $R_{\lambda }$. The dashed line in (*a*) indicates a power law of $\Sigma^{1/2}$. (Online version in colour.)

The power-law behaviour of $-\langle S_{ij}S_{jk}S_{ki}|\Sigma \rangle ∼\Sigma^{3/2}$ (for $\Sigma \tau_{K}^{2}>1$) suggests that the magnitude of individual eigenvalues of strain would simply scale as $\Sigma^{1/2}$. Figure 2*a* shows the conditional average of first two eigenvalues, and confirms this expectation (the third eigenvalue, which has the largest magnitude, can be obtained via the incompressibility condition). It can also be seen that $\lambda_{2}$ is always positive, but does not scale as $\Sigma^{1/2}$ for weak strain events ($\Sigma \tau_{K}^{2}<1$), and instead has a larger exponent. This can be explained by realizing that when the magnitude of strain approaches zero, $\lambda_{2}$ would also approach zero, due to strong cancellation between $\lambda_{1}$ and $\lambda_{3}$. This expectation is verified in figure 2*b*, which shows the quantity β [16,26], defined as

3.7 $$\beta =\sqrt{6}\frac{\lambda_{2}}{\sqrt{\lambda_{1}^{2}+\lambda_{2}^{2}+\lambda_{3}^{2}}}=\sqrt{12}\frac{\lambda_{2}}{\Sigma^{1/2}},$$

conditioned on Σ. It can be observed that β→0 when Σ→0, and only for $\Sigma \tau_{K}^{2}>1$, it becomes a constant (provided the $R_{\lambda }$ is sufficiently high). It is worth noting that this overall trend for $\lambda_{2}$ also explains the behaviour of strain self-amplification term in figure 1*a* for the region $\Sigma \tau_{K}^{2}<1$.

The vortex stretching term can be expressed in the eigenframe of strain as

**Figure 3.**
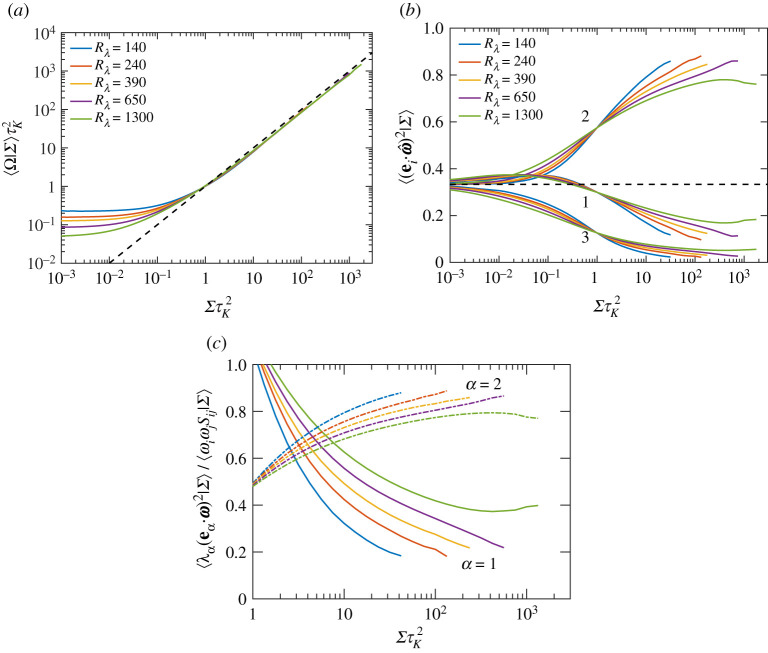
Conditional expectations (given Σ) of (*a*) enstrophy, Ω, (*b*) second moment of alignment cosines between vorticity and eigenvectors of strain, and (*c*) the relative contribution to the vortex stretching term from the first two eigendirections of strain, for various $R_{\lambda }$. The dashed line in (*a*) corresponds to a power-law of $\Sigma^{1}$. The dashed line in (*b*) is at 1/3, corresponding to a uniform distribution of alignment cosines (indicating lack of any preferential alignment). (Online version in colour.)

3.8 $$\omega_{i}\omega_{j}S_{ij}=\lambda_{i}{\left({\text{e}}_{i}\cdot \omega \right)}^{2}=\Omega \lambda_{i}{\left({\text{e}}_{i}\cdot \hat{\omega }\right)}^{2},$$

with $\hat{\omega }=\omega /|\omega |$, highlighting the importance of the alignment of vorticity with strain-eigenvectors (along with magnitude of vorticity and strain) in determining the efficacy of vortex stretching (or strain depletion in this case) [7,26]. The conditional expectation of enstrophy is shown in figure 3*a*, exhibiting the same qualitative behaviour as $\omega_{i}\omega_{j}S_{ij}$ in figure 1*a*. For intense strain events ($\Sigma \tau_{K}^{2}>1$), we find that ⟨Ω|Σ⟩∼Σ, suggesting a simple causal relation that intense strain produces equally intense vorticity (as anticipated from vortex stretching). On the other hand, for weak strain events ($\Sigma \tau_{K}^{2}<1$), the conditional average is constant, suggesting a lack of correlation between strain and vorticity [7,16] (which is also reflected in the behaviour of $\omega_{i}\omega_{j}S_{ij}$ in figure 1*a*).

Figure 3*b* shows the conditional expectation of the second moment of alignment cosines, i.e. $\left\langle {\left(e_{i}\cdot \hat{\omega }\right)}^{2}|\Sigma \right\rangle$, which are individually bounded between 0 and 1, respectively for orthogonality and perfect alignment, and are equal to 1/3 for no preferential alignment (corresponding to a uniform distribution of the cosine). Additionally, the three alignment cosines (for i=1,2,3) also add up to unity. Overall, the alignments follow the same trend as when conditioned on vorticity (see fig. 3*d* in [16]), i.e. in regions of intense strain ($\Sigma \tau_{K}^{2}>1$), vorticity is strongly aligned with $e_{2}$ and preferentially orthogonal to both $e_{1,3}$ (more so with $e_{3}$). Whereas for $\Sigma \tau_{K}^{2}\ll 1$, the alignments approach 1/3, reaffirming a lack of correlation between strain and vorticity. However, unlike when conditioned on vorticity (in [16]), the alignments in figure 3*b* show a significant $R_{\lambda }$-dependence. The emergence of a plateau-like behaviour for $R_{\lambda }=1300$ suggests an asymptotic state would likely be reached if $R_{\lambda }$ is further increased.

Figure 3*c* shows the relative contributions of each eigenvalue to the overall vortex stretching term. We only show contributions corresponding to first and second eigenvalues, which are both positive. The (negative) contribution for the third eigenvalue is quite small, and can be evaluated by realizing that all three contributions add up to unity. Interestingly, we note that the contribution from the second eigenvalue is significantly stronger than that from the first eigenvalue, and accounts for most of vortex stretching. The difference between the two gradually decreases with $R_{\lambda }$, but nevertheless, even at the highest $R_{\lambda }$ (=1300), the second eigenvalue contributes to nearly 80% of the net vortex stretching. The results on alignments in figure 3*b*, combined with these indicate a strong structural difference between regions of intense strain and vorticity. In regions of intense vorticity, even though vorticity is strongly aligned with the second eigenvector, the first eigenvalue contributes more significantly to overall vortex stretching [16]. This difference can be explained by realizing that the relative magnitude of $\lambda_{2}$ itself is significantly smaller in regions of intense vorticity (compared to regions of intense strain) [16].

From a structural point of view, the above results are consistent with the notion that intense vorticity is arranged in tube-like structures, whereas intense strain is arranged in sheet-like structures [20]. In both scenarios, vorticity has the propensity to align with the second eigenvector of strain. However, for the case of vortex tubes, the corresponding magnitude of the second eigenvalue is significantly smaller [16]. We will discuss more about this later in §3(c).

### (b) Role of pressure Hessian

**Figure 4.**
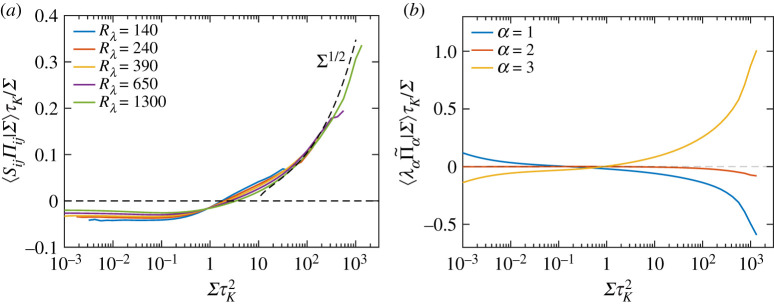
Conditional expectations (given Σ) of (*a*) strain and pressure-Hessian correlation for various $R_{\lambda }$, and (*b*) the individual contributions in the eigenframe of strain, as defined by equation (3.9), for only $R_{\lambda }=1300$. The expectations are once again divided by Σ, and all quantities are non-dimensionalized by $\tau_{K}$. (Online version in colour.)

Next, we look at the contribution of pressure Hessian to generation of strain. To this end, figure 4*a* shows the conditional average $\left\langle S_{ij}\Pi_{ij}|\Sigma \right\rangle$, once again divided by Σ for convenience. Since the corresponding unconditional average is zero, the conditional average cannot keep the same sign for all values of Σ. Figure 4*a* shows that for events stronger than the mean ($\Sigma \tau_{K}^{2}≳1$) this quantity is positive, and thus leads to depletion of strain (due to the negative sign associated with the term in equation (3.2)), and vice versa for events weaker than the mean. Thus, the non-local pressure field on average acts to redistribute the strain fluctuations towards its mean amplitude [27]. It should be further noted that, for intense strain, the conditional average scales once again as $\Sigma^{3/2}$, whereas for weak strain events it scales as $\Sigma^{1}$—albeit with a much smaller pre-factor (in both regimes) when compared with the strain self-amplification or vortex stretching terms.

While the overall contribution of the pressure field is to drive strain fluctuations towards the mean field, it is once again instructive to analyse the individual contributions in the eigenframe of strain tensor—where the strain-pressure Hessian correlation can be rewritten as

3.9 $$S_{ij}\Pi_{ij}=\lambda_{i}{\tilde{\Pi }}_{i},$$

where ${\tilde{\Pi }}_{\alpha }=e_{\alpha }^{T}\Pi e_{\alpha }$ is the projection of pressure Hessian tensor along the eigenvector $e_{\alpha }$ of the strain and repeated α does not imply summation (a convention which we will adhere to henceforth). Note that the eigenvalues of strain can also be defined in a similar way: $\lambda_{\alpha }=e_{\alpha }^{T}Se_{\alpha }$. We also introduce the eigenframe of the pressure Hessian tensor, defined by eigenvalues $\lambda_{i}^{p}$ (for i=1,2,3 and also arranged in descending order) and corresponding eigenvectors $e_{i}^{p}$, leading to

3.10 $${\tilde{\Pi }}_{i}=\lambda_{j}^{p}{\left({\text{e}}_{i}\cdot {\text{e}}_{j}^{p}\right)}^{2}$$

and

3.11 $$S_{ij}\Pi_{ij}=\lambda_{i}\lambda_{j}^{p}{\left({\text{e}}_{i}\cdot {\text{e}}_{j}^{p}\right)}^{2}.$$

Note $\sum_{\alpha }\lambda_{\alpha }^{p}=\nabla^{2}P/\rho =(\Omega -\Sigma )/2$ (using equation (1.3)). Thus, from the result in figure 3, i.e. $\langle \Omega |\Sigma \rangle ≃c\Sigma^{1}$ (with c≲1), it follows that in regions of intense strain the sum of three eigenvalues is overall small in magnitude with a negative sign—which in turn suggests a dominant role of $\lambda_{3}^{p}$, which we analyse next.

Figure 4*b* shows the breakup of individual contributions to strain-pressure Hessian correlation as given in equation (3.10), i.e. $\left\langle \lambda_{\alpha }{\tilde{\Pi }}_{\alpha }|\Sigma \right\rangle$ (we recall again that no summation is implied over α). It is observed that the dominant positive contribution comes from the third eigenvalue of strain (and hence leads to depletion of strain), whereas the other two contributions are negative (leading to amplification of strain). These trends can be simply explained from equation (3.11) by assuming that the alignments ${\left(e_{i}\cdot e_{j}^{p}\right)}^{2}$ are all 1/3 (corresponding to lack of any no preferential alignment)—leading to $\lambda_{\alpha }\tilde{\Pi_{\alpha }}=\lambda_{\alpha }\sum_{\alpha }\lambda_{\alpha }^{p}=\lambda_{\alpha }\nabla^{2}P/\rho$. Since $\nabla^{2}P$ is slightly negative for $\Sigma \tau_{K}^{2}>1$, it follows that $\lambda_{\alpha }\tilde{\Pi_{\alpha }}$ has the opposite sign as that of $\lambda_{\alpha }$, consistent with figure 4*b*.

**Figure 5.**
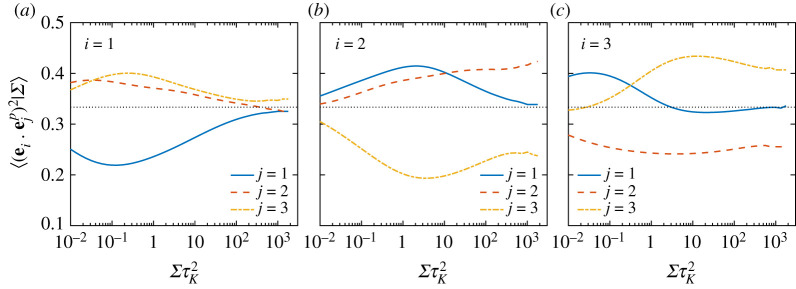
Conditional expectations (given Σ) of the alignment cosines between eigenvectors of strain and pressure Hessian, respectively, $e_{i}$ and $e_{j}^{p}$. (Online version in colour.)

An important assumption in the argument above was the lack of any preferential alignment between the eigenvectors of strain and those of pressure Hessian. Figure 5 shows the conditional second moments of various alignment cosines, i.e. $\left\langle {\left(e_{i}\cdot e_{j}^{p}\right)}^{2}|\Sigma \right\rangle$. Note that, similar to alignment cosines between vorticity and strain, the alignment cosines for strain and pressure Hessian for any fixed value of i (or j) add up to unity. Additionally, they are all individually bounded between 0 and 1, for orthogonal and perfect alignment respectively; whereas for non-preferential alignment the averages would be 1/3 (corresponding to uniform random distribution). Figure 5 reveals that the deviation of all the alignments from 1/3 is very small, therefore excluding any strong alignments between the two sets of eigenvectors. This is to be contrasted with the strong alignment observed between vorticity and strain in figure 3*b*. It is worth noting that conditioning the alignment properties on $Q=\frac{1}{2}(\Omega -\Sigma )$, the second invariant of velocity gradient tensor suggests a somewhat stronger alignment between the eigenvectors of strain and pressure-Hessian for the strain-dominated regions, i.e. Q<0 [28]. However, the strain-dominated regions as identified by Q<0 are not the same as those identified here by directly using the magnitude of Σ. This aspect warrants a separate investigation.

### (c) Budget of nonlinear terms and strain decomposition

**Figure 6.**
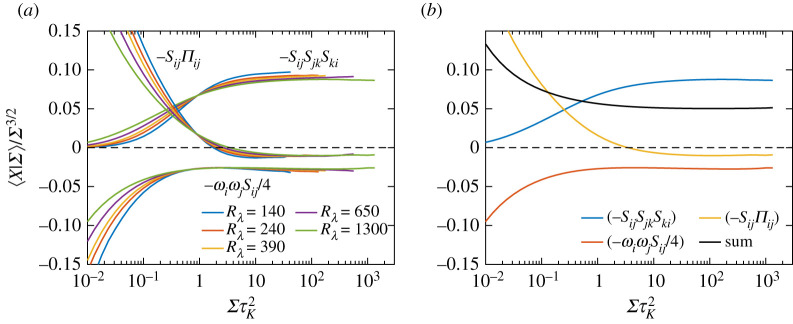
(*a*) Conditional expectations (given Σ) of various nonlinear (inviscid) terms on the r.h.s. of equation (3.2), for various $R_{\lambda }$. All quantities are normalized by $\Sigma^{3/2}$ revealing a plateau like behaviour for $\Sigma \tau_{K}^{2}>1$. The same quantities are shown in (*b*) for $R_{\lambda }=1300$, to highlight the combined contributions of the terms. (Online version in colour.)

Following upon the results in previous subsections, figure 6*a* compares the contributions of various nonlinear (inviscid) terms on the r.h.s. of equation (3.2) (note that the viscous term is simply the negative of the net contribution of all the inviscid terms). All the terms are now normalized by $\Sigma^{3/2}$, and clearly show a plateau for $\Sigma \tau_{K}^{2}>1$. As expected, the dominant positive contribution comes from the strain self-amplification term, whereas the vortex stretching term is negative and significantly smaller in magnitude. The contribution from pressure Hessian term is also negative for $\Sigma \tau_{K}^{2}≳1$ and even smaller in magnitude. To get more insight into the balance of terms in equation (3.2), figure 6*b* shows the data only for $R_{\lambda }=1300$, including the resulting sum between various terms.

While the overall contribution of various inviscid terms in generating intense strain, as illustrated in figure 6, was relatively straightforward, a more complex picture emerges when considering amplification of individual eigenvalues of strain. To this end, we consider the transport equation for each eigenvalue [29,30]:

3.12 $$\frac{D\lambda_{\alpha }}{Dt}=-\lambda_{\alpha }^{2}+\frac{\Omega }{4}[1-{\left({\text{e}}_{\alpha }\cdot \hat{\omega }\right)}^{2}]-{\tilde{\Pi }}_{\alpha }+\text{viscous term}.$$

Multiplying both sides by $\lambda_{\alpha }$ leads to the equation

3.13 $$\frac{1}{2}\frac{D\lambda_{\alpha }^{2}}{Dt}=-\lambda_{\alpha }^{3}+\frac{1}{4}\Omega \lambda_{\alpha }[1-{\left({\text{e}}_{\alpha }\cdot \hat{\omega }\right)}^{2}]-\lambda_{\alpha }{\tilde{\Pi }}_{\alpha }+\text{viscous term}$$

providing the individual breakups for equation (3.2), since $\Sigma =2\sum_{\alpha }\lambda_{\alpha }^{2}$. Note that the individual eigenvalues now have a direct contribution from vorticity, from the term $\Omega \lambda_{\alpha }$, which sums up to zero in equation (3.2) due to incompressibility. This leads to a more involved interplay between strain self-amplification and vortex stretching at the level of individual eigenvalues, than for the total strain in figure 6.

**Figure 7.**
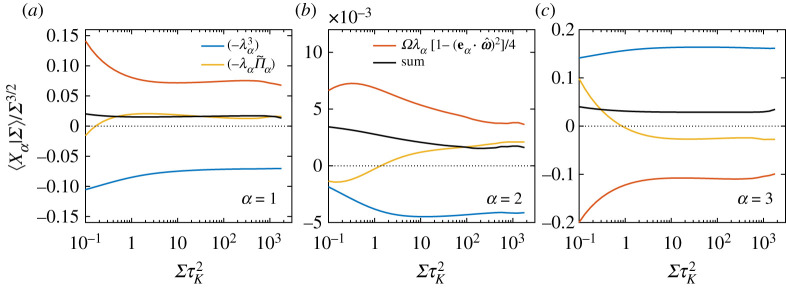
Conditional expectations of the various inviscid terms in equation (3.13) for (*a*) i=1, (*b*) i=2, and (*c*) i=3 (note that repeated indices do not imply summation). All quantities have been normalized by $\Sigma^{3/2}$. The legend in (*a*,*b*) is common to all figures. (Online version in colour.)

Since the first two eigenvalues of strain, $\lambda_{1}$ and $\lambda_{2}$, are positive, it follows that the self-amplification term $-\lambda_{\alpha }^{3}$ leads to depletion instead of actual amplification, whereas the contribution due to vortex stretching is overall positive and leads to amplification (since $1-{\left(e_{\alpha }\cdot \hat{\omega }\right)}^{2}>0$). By contrast, for $\lambda_{3}$, which is negative, the amplification originates from $-\lambda_{3}^{3}$, and vortex stretching leads to depletion. The sign of the pressure Hessian term, $-\lambda_{\alpha }{\tilde{\Pi }}_{\alpha }$ will also be the same as that of $\lambda_{\alpha }$ (as shown in figure 4), and thus would amplify $\lambda_{1}$ and $\lambda_{2}$, but deplete $\lambda_{3}$. These expectations are all qualitatively confirmed in figure 7, which shows the conditional averages of various terms for each eigenvalue, conditioned on Σ and normalized by $\Sigma^{3/2}$. Note that the individual contributions shown in figure 7*a*–*c* sum up to the terms shown in figure 6*b*. Quantitatively, we find two main trends. For the case of $\lambda_{1}$ and $\lambda_{2}$, in figure 7*a*,*b* respectively, the contributions from self-amplification and vortex stretching terms approximately cancel each other out (for $\Sigma \tau_{K}^{2}>1$), and the net nonlinear amplification almost entirely results from the pressure Hessian term. For $\lambda_{3}$, in figure 7*c*, there is significant cancellation between the self-amplification and vortex stretching terms (and the pressure Hessian term now aids in depletion), but the self-amplification overall dominates.

Thus, the following picture of strain amplification emerges. The self-amplification of strain only occurs along the third eigendirection, i.e. via self-compressive motion, whereas the same mechanism depletes the first two eigenvalues. On the other hand, as strain acts to amplify vorticity (via vortex stretching), the feedback leads to amplification of the first two eigenvalues (which could further aid in vorticity amplification), but leads to depletion of the third eigenvalue. The net result is such that these two effects nearly balance each other for the first two eigenvalues, but the self-amplification prevails for the third eigenvalue. Thus, overall these terms only act to make $\lambda_{3}$ more negative (and thus produce strong compressive motion). In this context, the pressure Hessian term acts to deplete the third eigenvalue, and in turn amplify the first two, qualitatively producing a similar effect to vortex stretching.

These results clearly demonstrate that the generation of intense strain differs in crucial aspects compared to the generation of intense vorticity [7,16]. While vortex stretching solely enables generation of intense vorticity, it also acts to deplete strain at the same spatial location. This suggests that the local maximas of vorticity and strain are never likely to be colocated, which has been confirmed in DNS [5,20,21] and also corroborated by a simple vortex tube calculation [23]. At the same time, regions of intense strain and vorticity also have to be sufficiently ‘nearby’ and correlated, since strain and vorticity are coupled via the Biot–Savart relation [14,15]. This is also consistent with observations from DNS, which show that intense strain is arranged in sheet-like structures neighbouring tube-like regions of intense vorticity [5]. Finally, these structural differences are in turn consistent with how vorticity-strain correlations differ between regions of intense strain and vorticity. Thus, analysing the non-local relation between strain and vorticity could be vital to understand their amplification [15,17] and also could provide a quantitative reasoning as to why vorticity is more intermittent than strain [5]. It would also be worthwhile to obtain the structure of regions of intense vorticity and strain in the eigenframe of strain, by appropriately extended the averaging procedure introduced in [31], which does not resort to any simplifying approximation on the structure of the underlying structures.

## 4. Conclusion

In this work, we have used a massive DNS database of stationary isotropic turbulence with Taylor-scale Reynolds number in the range 140–1300 to analyse the nonlinear mechanisms responsible for generation of extreme events of energy dissipation (and hence strain rate), identified by $\Sigma =2S_{ij}Sij$, where $S_{ij}$ is the strain-rate tensor. We have investigated the three nonlinear processes involved in the transport equation for Σ (see equation (3.2)), viz., the strain self-amplification, vortex stretching and strain-pressure Hessian correlation, by analysing their statistics conditioned on Σ. We find that the overall amplification of strain comes from the strain-self amplification term only, whereas the other two terms act to deplete intense strain events. Remarkably, the dependence of various conditional averages on Σ follows a simple dimensional consideration.

The three mechanisms are further analysed in the eigenbasis of strain tensor, defined by its eigenvalues $\lambda_{i}$ and eigenvectors $e_{i}$ (for i=1,2,3), revealing a more complex picture. Since $\lambda_{1}$ is always positive and $\lambda_{2}$ is positive on average, it follows that the self-amplification term in fact leads to depletion for these eigenvalues, whereas the vortex stretching and pressure Hessian terms lead to amplification. Surprisingly, the self-amplification and vortex stretching terms cancel each other and the net amplification is solely provided by the pressure Hessian term. By contrast, the behaviour of all these terms for the third eigenvalue (which is always negative) is similar to that of total strain, revealing that extreme events of strain result from strong self-compressive action. Our results are consistent with the notion that regions of intense strain are arranged in sheet-like structures, in the vicinity of, but never colocated with regions of tube-like intense vorticity [5,20,21,23]. In this context, analysing the non-local relation between strain and vorticity would be crucial in understanding their amplification [14,15,17] and could also shed light on the long-standing mystery of why vorticity is more intermittent than strain.

Finally, we note that the conditional statistics investigated in this work have very simple power-law dependencies on Σ (in the region Σ/⟨Σ⟩>1), as deducible from an elementary dimensional consideration. We have listed all such relevant quantities in table 2, which could be valuable in statistical modelling of energy dissipation rate, especially in PDF methods [32]—an exercise left for future work.

**Table 2.** Prefactors $c_{X}$ for the asymptotic scaling of various conditional statistics satisfying $\langle X|\Sigma \rangle \approx c_{X}\Sigma^{p}$ (in the range Σ/⟨Σ⟩>1), such that $X/\Sigma^{p}$ is dimensionless $\lambda_{i}$ and ${\text{e}}_{i}$ are the eigenvalues and eigenvectors of strain tensor, respectively. ω is the vorticity and $\Omega =\omega_{i}\omega_{i}$. $\Pi_{ij}$ is the pressure Hessian tensor, and ${\tilde{\Pi }}_{i}$ is its projection along $e_{i}$. The results are obtained from the $R_{\lambda }=1300$ data.

X	$c_{X}\Sigma^{1/2}$	X	$c_{X}\Sigma^{1}$	X	$c_{X}\Sigma^{3/2}$	X	$c_{X}\Sigma^{3/2}$	X	$c_{X}\Sigma^{3/2}$
$\lambda_{1}$	0.412	$\lambda_{1}^{2}$	0.173	$\lambda_{1}^{3}$	0.074	$\lambda_{1}{\left({\text{e}}_{1}\cdot \omega \right)}^{2}$	0.042	$\lambda_{1}{\tilde{\Pi }}_{1}$	−0.013
$\lambda_{2}$	0.138	$\lambda_{2}^{2}$	0.024	$\lambda_{2}^{3}$	0.004	$\lambda_{2}{\left({\text{e}}_{2}\cdot \omega \right)}^{2}$	0.090	$\lambda_{2}{\tilde{\Pi }}_{2}$	−0.002
$\lambda_{3}$	−0.550	$\lambda_{3}^{2}$	0.303	$\lambda_{3}^{3}$	−0.167	$\lambda_{3}{\left({\text{e}}_{3}\cdot \omega \right)}^{2}$	−0.019	$\lambda_{3}{\tilde{\Pi }}_{3}$	0.024
		Ω	0.856	$S_{ij}S_{jk}S_{ki}$	−0.089	$\omega_{i}\omega_{j}S_{ij}$	0.113	$S_{ij}\Pi_{ij}$	0.010

## Data accessibility

The data that support the findings of this study are available from the corresponding author on request.
